# LTiT: A Deep Learning Model for Subway Section Passenger Flow Prediction Based on LSTM-TSSA-iTransformer

**DOI:** 10.3390/s26092584

**Published:** 2026-04-22

**Authors:** Jie Liu, Yanzhan Chen, Yange Li, Fan Yu

**Affiliations:** School of Traffic and Transportation Engineering, Central South University, Changsha 410075, China234201019@csu.edu.cn (Y.L.); yufan9705@163.com (F.Y.)

**Keywords:** subway section passenger flow, LSTM, TSSA, iTransformer

## Abstract

As a vital part of urban public transportation system, subway passenger flow prediction plays a crucial role in alleviating traffic congestion, improving transportation infrastructure, and optimizing travel experience. Existing subway passenger flow prediction mainly focuses on short-term predictions of inbound/outbound passenger flow and origin-destination (O-D) demand. Subway section passenger flow prediction can provide a more direct reflection of passenger fluctuations across different line segments, and offer robust support for management and resource allocation. We propose a subway section passenger flow generation model and a prediction method based on LTiT (LSTM-TSSA-iTransformer). This model is based on the overall architecture of the iTransformer encoder, and an LSTM (Long Short-Term Memory) network is employed to capture the temporal characteristics of subway section passenger flow. This is combined with the TSSA (Token Statistics Self-Attention) to adaptively weight the information at key time points. Efficient performance of the model was evaluated by comparing its predictions with other models, including SARIMA (Seasonal Auto-Regressive integrated moving average), BP neural networks, LightGBM (Light Gradient Boosting Machine) and LSTM (Long Short-Term Memory). Experimental results show that the proposed model outperforms traditional baseline models in evaluation metrics such as R^2^, MAE, MSE, and MAPE. Finally, we further investigate the selection of input window length and prediction step size, and perform robustness analysis under different noise conditions.

## 1. Introduction

Continuous development of urban rail transit has led to an increasingly dense network structure. Rail transit has become backbone of urban public transportation and main way for citizens to travel, providing services for daily commuting and travel of passengers. In recent years, concepts of smart cities and intelligent transportation have shown significant development trends, driving rail transit towards more advanced and intelligent directions. The Automatic Fare Collection system (AFC), collecting data from RFID/NFC and infrared sensors, enables operators to monitor passenger flow in subway stations in real-time, accurately track entry and exit information, and predict future trends in ridership. Real-time monitoring of subway section passenger flow enables the early identification of future passenger patterns. It can help operators grasp occupancy status of lines, optimize subway scheduling plans, improve operational efficiency and service quality, provide decision support for urban transportation planning, and improve travel experience.

Traditional parameter prediction models use time series analysis methods for prediction. The Kalman Filtering method minimizes variance, maps regression problem to state space to find optimal solution, and then uses real-time prediction error to correct randomness and improve accuracy [1,2]. The Autoregressive Integrated Moving Average model (ARIMA) uses differential methods to eliminate data instability and construct a stationary sequence containing autoregressive (AR) and moving average (MA) components for prediction [3]. Although random prediction models have high computational efficiency under specific conditions, they often struggle to capture nonlinear and dynamic characteristics of traffic flow data. Consequently, models combining Kalman Filter models with radial basis function neural networks and series models derived from ARIMA have emerged to overcome the low prediction accuracy of individual models for short-term traffic flow forecasting [4,5,6]. The Seasonal Autoregressive Integrated Moving Average (SARIMA) model, as an ex-tension of the ARIMA model, accounts for seasonal factors and captures both seasonal fluctuations and long-term trends in the data, making it widely used in time series analysis [7]. In recent years, the research on SARIMA hybrid models has gradually in-creased. The Enhanced TimesNet-SARIMA Model, by decomposing the data into Intrinsic Mode Functions (IMFs) and combining TimesNet with SARIMA for adaptive forecasting, has shown significant improvements in predicting subway outbound flow during peak hours [8].

To further address the high volatility and random noise in traffic flow, numerous studies have introduced signal decomposition and multi-model ensemble techniques. The Sparrow Search Algorithm (SSA) and optimized Variational Mode Decomposition (VMD) technique can adaptively decompose the original sequence into multiple subsequences, which are then combined with traditional statistical models such as Bi-GRU [9], or Graph Attention Networks (GAT) [10] for hybrid forecasting. This decompose-then-forecast strategy shows remarkable results when handling passenger flow components of different frequencies [11]. Additionally, Gradient Boosting Decision Trees (GBDT) have been effectively used to quantify the impact of external factors, such as bus connections, on short-term passenger flow [12]. Timestamp-guided knowledge distillation frameworks [13] and lightweight cross-linear models designed for Industrial Internet of Things [14] also provide insightful cross-domain solutions for traffic flow forecasting.

To further extract meaningful insights from time series data and improve the accuracy of forecasting, deep learning has been extensively applied in traffic flow prediction [15,16,17]. Recurrent Neural Networks (RNNs) and their variants play a significant role in the field of traffic flow forecasting. Researchers use autoencoders to extract upstream and downstream traffic flow features before inputting them into LSTM, effectively handling complex linear traffic flow data [18,19]. By utilizing deep learning models with different temporal features, efficiency can be significantly improved and performance of model can be enhanced. The LSTM-CNN model uses oversampling techniques to learn various features, such as traffic flow characteristics, signal timing, and weather conditions, to predict traffic flow on urban main roads [20]. T-LSTM model takes into account daily periodicity of traffic flow and predicts traffic flow of a single road segment [21]. Targeted optimization of the hybrid LSTM structure for different traffic conditions can further improve the real-time performance and accuracy of predictions [22]. To overcome the problem of traditional networks falling into local optima, researchers introduced an improved Whale Optimization Algorithm to optimize the RNN parameters, significantly enhancing the model’s robustness [23]. Combining Time Convolutional Networks with LSTM and incorporating attention mechanisms to handle external factors has achieved high accuracy in origin-destination (OD) passenger flow prediction [24]. In short-term subway passenger flow prediction, the construction of dynamic time-varying networks and the integration of the TSF-GRU model successfully captured the joint influence of temporal dependence, spatial correlation, and network topology [25]. In addition to data-driven statistical and machine learning methods, theory-based traffic models have provided an important way to describe traffic dynamics. Using macroscopic traffic flow modeling and identifying key parameters from observed data, can verify the model’s ability to describe traffic flow evolution [26]. It plays an important role in the practical application of the model. A notable advancement is the development of macroscopic traffic flow model-integrated deep learning frameworks. These methods bring together temporal-spatial traffic dependency, traffic flow theory, and data analysis techniques [27]. In this way, they can better describe the dynamic spread of traffic states across road networks. It is important to capture the inherent structural characteristics and dynamic patterns of traffic networks.

As the research perspective shifts from a single time dimension to the complex network spatial topology, Graph Neural Networks (GNNs) have gradually become a key solution to address spatial heterogeneity. The topology of modern transportation networks has profound and dynamic evolution effects, prompting frontier research to overcome the limitations of static adjacency matrices. For instance, by constructing a dynamic sparse graph convolution model combined with GRU, it effectively simulates information diffusion within dynamic spatial structures [28]. The two stage pre-trained trend-aware dynamic graph learning framework better extracts spatial topology and local trends [29]. Additionally, the Spatio-Temporal Heterogeneity-Oriented Graph Convolutional Network innovatively integrates urban street differences and external cross-domain factors, such as air quality [30]. This graph network concept not only applies to ground and rail traffic but also demonstrates powerful feature extraction and generalization capabilities in airport topology networks in the aviation field [31] and multimodal traffic flow prediction for terminal areas considering complex weather conditions [32].

Transformer uses self-attention mechanisms to replace recurrent neural networks and convolutional neural networks. It can demonstrate superior performance across computer vision, natural language processing, and other domains [33,34,35,36]. However, researchers have found that, in certain temporal tasks, Transformer exhibits limited ability to capture time-series features and multivariate correlations. To overcome these limitations, recent studies have made targeted improvements to the Transformer architecture. Some introduce multi-granularity temporal embedding to reduce the loss of historical time information [37]. In the patch mechanism, frequency compensation is incorporated using Fast Fourier Transform such as FCP-Former to mitigate the loss of temporal information within blocks [38]. Complex Transformer may perform worse than simple linear layers in terms of both performance and efficiency [39]. iTransformer [40] treats each time series as a variable token, employs an attention mechanism to capture multivariate correlations, and uses a feedforward network for sequence representation. This approach effectively addresses the limitations of Transformer-based prediction models. iTransformer-FFC incorporates the Fast Fourier Convolution module, isolating periodic components and attenuates noise in the face of multi-scale and highly non-stationary sequences [41].

With the rapid expansion of urban rail transit systems, subways have become the backbone of modern urban public transportation. Subway passenger flow prediction is crucial for optimizing traffic management, adjusting train schedules, improving operational efficiency, and enhancing the passenger travel experience. Traditional subway passenger flow forecasting primarily focuses on inbound/outbound station flow prediction [42,43,44] and origin-destination (OD) flow prediction [45,46,47,48]. While such studies help subway operators optimize station flow management, they often fail to effectively reflect the dynamic changes in passenger flow across different sections.

Subway section passenger flow prediction is more complex and challenging. Prediction passenger flow at the section level can directly reflect passenger fluctuations across different line segments, helping subway operators carry out more precise resource allocation and train headway adjustment. Although section-level passenger flow forecasting has substantial practical value, research in this area remains relatively limited. Traditional linear statistical models such as ARIMA [49,50], SARIMA [51,52], and various deep learning models, have demonstrated a certain degree of effectiveness in short-term passenger flow prediction. Traditional models rely on linear assumptions and inherently struggle to capture the non-stationary and highly nonlinear dynamic changes characteristic of subway section passenger flow. While deep learning models are well-equipped to handle nonlinear complexities, current methods often struggle to identify important time points and random noise. To address this issue, we propose a subway section passenger flow prediction method based on subway AFC system data and timetable, with the aim of overcoming the limitations of existing research, directly reflecting the level of subway congestion, and improving the accuracy and reliability of subway section passenger flow forecasting. The contributions of this paper are as follows:

➢ A method for calculating subway section passenger flow based on AFC passenger entry/exit data, and subway schedules is proposed. Unlike traditional station-level prediction, this approach provides interval-based flow forecasting, offering precise decision support for subway operational resource scheduling and line optimization. Additionally, we incorporate external variables such as temperature and precipitation into the data. It provides new data support for passenger flow prediction.
➢ An LTiT prediction model was designed. This model uses LSTM to capture the temporal characteristics of subway section passenger flow, and uses TSSA to identify high-information time periods. The iTransformer architecture was employed to effectively learn these temporal features. By combining these strengths, the model better handles complex temporal data and adapts to the impact of external variables on passenger flow.
➢ We conducted comparative experiments between the LTiT model and traditional benchmark models on both high-flow section and low-flow section. The results show that the LTiT model significantly outperforms other models in evaluation metrics such as R^2^, MAE, MSE, and MAPE, particularly in tasks involving subway section passenger flow forecasting with complex time series and external variable influences, demonstrating higher accuracy and stability.

## 2. Methodology

### 2.1. Problem Definition and Framework

Forecasting subway section passenger flow helps operators monitor real-time car load conditions. By predicting passenger volumes across different sections, operators can adjust train intervals or departure times for specific segments in advance based on the forecasts. Generally speaking, the subway transit data we obtain is recorded by the subway AFC system as entry and exit information (including records of single-trip tickets, physical cards, and internet tickets). This data essentially represents passengers’ OD trip records. We combine train timetables with line topology rules to map passenger trips to the actual operating sections they traverse, and then aggregates this data at fixed time intervals to generate sectional passenger flow sequences.

Consider a subway network composed of a set of directed intervals. Let the interval set be defined as:

(1) $$S=\{s_{1},s_{2},\ldots ,s_{\left|S\right|}\}$$

Each interval s=(u→v) represents the operating section between adjacent stations u and v. By discretizing continuous time into equally spaced time points t=1,2,⋯,T, the passenger flow through section s at time point t is defined as the number of passengers passing through the section during that time period, ${\mathbb{R}}_{\geq 0}$ represents non-negative real numbers:

(2) $$x(t)≜x_{s}(t)\in {\mathbb{R}}_{\geq 0}$$

The resulting time series for a single section is:

(3) X={x(1),x(2),…,x(T)}

The prediction task can be defined as follows: given the subway section passenger flow at L past time points, predict the cross-sectional passenger flow at H future time points.

Set the historical L input window as:

(4) $$x_{t-L+1:t}={\left[x(t-L+1),\ldots ,x(t)\right]}^{⊤}\in {\mathbb{R}}^{L}$$

Set H prediction target as:

(5) $${\hat{x}}_{t+1:t+H}={\left[\hat{x}(t+1),\ldots ,\hat{x}(t+H)\right]}^{⊤}\in {\mathbb{R}}^{H}$$

Additionally, to improve the prediction of the target, this study incorporates exogenous variables (such as temperature and precipitation), denoted as:

(6) $$P(t)\in {\mathbb{R}}^{d_{x}},P_{t-L+1:t}={\left[p(t-L+1),\ldots ,p(t)\right]}^{⊤}\in {\mathbb{R}}^{L\times d_{q}}$$

This prediction problem can be formalized as learning a mapping function from input to output $f\left(\cdot \right)$:

(7) $${\hat{x}}_{t+1:t+H}=f\;\left(x_{t-L+1:t},p_{t-L+1:t}\right)$$

**Figure 1:**
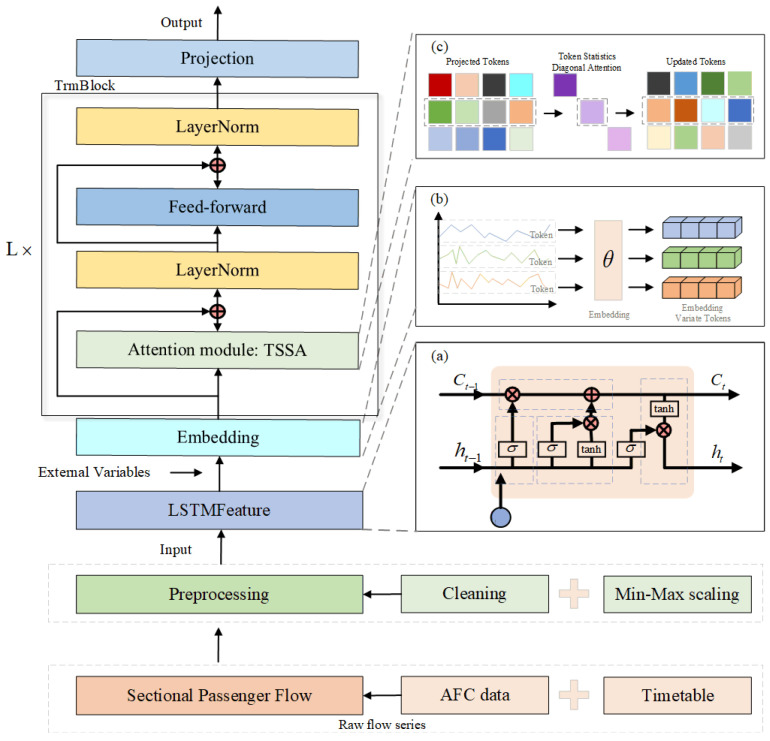
Overall structure of LTiT, which adopts the iTransformer architecture. (**a**) the traditional LSTM architecture; (**b**) the independent embedding of raw sequences from different variables as tokens; (**c**) TSSA computes statistical features for each token and adjusts the attention distribution via diagonal attention.

In Figure 1, we map passenger journeys to target sections s based on AFC entry/exit records, integrated with line topology and train schedules, and aggregates them into cross-sectional passenger flows at fixed time intervals x(t). After that, we perform data cleaning, normalization, and other preprocessing steps. The model adopts an iTransformer encoder architecture: input sequences are first normalized to mitigate the non-stationary effects caused by trend components and distribution drift in passenger flow sequences. Subsequently, the standardized sequence is fed into LSTM to extract local temporal features, which are then combined with external variables through embedding to integrate them into the sequence. Replacing the encoder’s original self-attention mechanism with TSSA, enables interaction through second-order moments based on token features, rather than calculating similarity between every token pair. This approach helps capture high-information segments within sequences, enhancing the model’s sensitivity to key features. The sequence processed by TSSA is then fed into the iTransformer encoder to model global relationships. Finally, the future prediction step H is obtained and back-normalized to yield ${\hat{x}}_{t+1:t+H}$.

### 2.2. LSTM: For Temporal Representation

**Figure 2:**
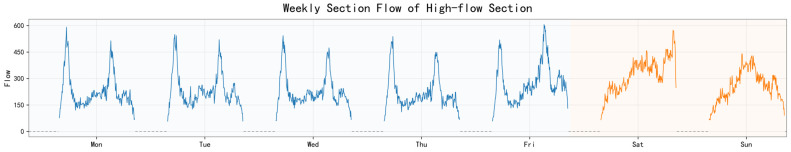
Weekly Section Flow of High-flow Section. The blue part indicates weekday section passenger flow variation, and the orange part indicates weekend section passenger flow variation.

**Figure 3:**
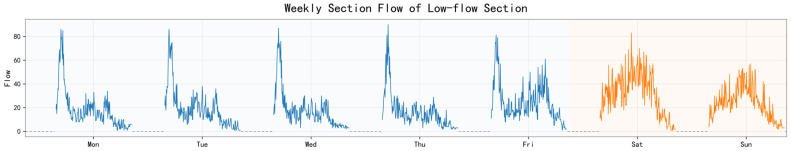
Weekly Section Flow of Low-flow Section. The blue part indicates weekday section passenger flow variation, and the orange part indicates weekend section passenger flow variation.

Subway section passenger flow exhibits stable intraday patterns and weekly cycles. Figure 2 and Figure 3 show the two typical types of section flow curves in the Changsha Metro sections. In high-flow sections, peak periods from Monday to Friday are pronounced and highly repetitive, with Friday evening rush hours showing higher peaks and greater fluctuations compared to Monday through Thursday. Weekend subway section passenger flow levels generally rise compared to weekdays, exhibiting more continuous and dispersed fluctuations. Saturday flows typically exceed Sunday’s intensity, while Sunday evening flows decline more sharply due to proximity to the workweek. In low-flow sections, weekday flow shows an extremely pronounced and singular unimodal peak pattern. Friday evening rush hours showing higher flow level, too. On weekends, as commuting ceases to be the primary driving force, this unimodal peak disappears, and the data curve instead presents a low, broad peak pattern that is flatter, wider, and accompanied by random fluctuations. These characteristics impart strong nonlinearity and temporal dependency to the sequence at local timescales. Therefore, we introduce an LSTM module to capture the temporal features of subway section passenger flow through its gated memory mechanism.

Perform Min-Max Scaling on each $x_{t}$:

(8) $${\tilde{x}}_{t}=\frac{x_{t}-\min(x)}{\max(x)-\min(x)}$$

where $x_{t}$ is the input value, min(x) and max(x) are the minimum and maximum values of the input data.

Let the normalized input sequence be:

(9) $$\tilde{X}=\{{\tilde{x}}_{1},{\tilde{x}}_{2},\ldots ,{\tilde{x}}_{L}\}\in {\mathbb{R}}^{B\times L\times N}$$

where B is the batch size, L is the input window length, and N is the number of input variables. The LSTM module employs a unidirectional multi-layer LSTM, whose gate updates at time step t can be expressed as:

(10) $$i_{t}=\sigma (W_{i}[h_{t-1},{\tilde{x}}_{t}]+b_{i})$$

(11) $$f_{t}=\sigma (W_{f}[h_{t-1},{\tilde{x}}_{t}]+b_{f})$$

(12) $$o_{t}=\sigma (W_{o}[h_{t-1},{\tilde{x}}_{t}]+b_{o})$$

(13) $${\tilde{c}}_{t}=\tanh(W_{c}[h_{t-1},{\tilde{x}}_{t}]+b_{c})$$

(14) $$c_{t}=f_{t}⊙c_{t-1}+i_{t}⊙{\tilde{c}}_{t}$$

(15) $$y_{t}=o_{t}⊙\tanh(c_{t})$$

where $i_{t}$, $f_{t}$, $o_{t}$ represent the input gate, forget gate, and output gate; $c_{t}$ denotes the memory unit state; $y_{t}$ represents the hidden state; $\sigma \left(\cdot \right)$ indicates the Sigmoid function; and ⊙ signifies element-wise multiplication.

To enhance the reproducibility and stability of the training process, the LSTM module uses a zero-initialization state for each batch:

(16) $$h_{0}=0,c_{0}=0$$

After multiple layers of recursion, the hidden sequence output is:

(17) $$Y=\{y_{1},y_{2},\ldots ,y_{L}\}\in {\mathbb{R}}^{B\times L\times d}$$

This sequence will be fed into the subsequent embedding layer, allowing the iTransformer encoder to learn smoother, higher-order feature representations. Such representations facilitate the modeling of intra-week periodic patterns and cross-period dependencies over extended temporal horizons.

### 2.3. Inspired by TSSA: Attention Module

Traditional self-attention mechanisms rely on pairwise token similarity to generate weights, and its computation and storage overhead increases quadrate with the number of tokens. TSSA avoids computing pairwise token similarity [53]. It constructs token updates based on statistical properties of token representations—especially the projected second-order moments—thereby achieving linear time and linear memory complexity.

In a subway section passenger flow prediction task, the contributions of different time points within the input window to the future flow are significantly different. Sudden changes during morning and evening peak hours, accumulation before peaks, and decline after peaks usually have more information. At the same time, passenger flows exhibit temporal characteristics and random noise. To enable the model to selectively emphasize key time points and suppress noise, we introduce a variant of TSSA after the embedding layer. This mechanism adaptively weights the representation of each time token, highlighting high information segments and weakening noise or redundancy, thereby improving the learning capability of iTransformer module.

The hidden sequence $Y=\{h_{1},h_{2},\ldots ,h_{L}\}\in {\mathbb{R}}^{B\times L\times d}$, produced after multiple layers of recursion through the LSTM module, is fed into the Embedding module and mapped to a d-dimensional embedding space. The resulting embedded tokens are:

(18) $$Z=\mathrm{Embedding}(Y)\in {\mathbb{R}}^{B\times L\times d}$$

where B is the batch size, L is the input window length, and d is the embedding dimension. The embedding of external variables is mapped through a linear layer to ensure consistency with the embedding dimension d of the time series data. In this way, we ensure that external variables and time series data are learned in the same embedding space. The concatenated data $Z^{\prime }$ will be passed as input to the subsequent TSSA module for processing. Based on $Z^{\prime }$, perform linear mapping and divide into multiple heads:

(19) $$W=\mathrm{reshape}(\mathrm{Linear}(Z))\in {\mathbb{R}}^{B\times K\times L\times d_{k}},\;\;\;\;\;\;\;\;\;\;d_{n}=d/N$$

where N denotes the number of heads. To characterize the information content of a token in different head subspaces at a given time step, compute the second-order moment for each token under each head:

(20) $$M_{b,k,l}=\sum_{i=1}^{d_{k}}{\tilde{w}}_{b,k,l,i}^{2},\;\;\;\;\;b\in \{1,\ldots ,B\},\;k\in \{1,\ldots ,K\},\;l\in \{1,\ldots ,L\}$$

where b represents the batch size, k corresponds to the head index, l denotes the token index at a particular time step. To enable the model to adaptively select among different heads at the same time point, a trainable temperature parameter $t_{h}$ is further introduced. A softmax operation is applied across the head dimension to obtain the soft allocation probability of tokens across different heads. The rationale for using the softmax function is to normalize the raw attention scores, ensuring that they are bounded between 0 and 1 and sum to 1, which makes them interpretable as probabilities. This allows the model to focus on the most informative tokens while suppressing less relevant ones. Additionally, the softmax function is differentiable, enabling the model to be trained end-to-end through backpropagation.

(21) $$\Pi_{b,k,l}={\mathrm{Softmax}}_{k}\left(t_{k}M_{b,k,l}\right)$$

To achieve adaptive adjustment of channels within the head, suppress random fluctuations and redundant components, and enhance effective information derived from structural variations, define a channel-by-channel gating function $G_{b,k,i}$ as:

(22) $$S_{b,k,i}=\sum_{l=1}^{L}{\hat{\Pi }}_{b,k,l}w_{b,k,l,i}^{2}$$

(23) $$G_{b,k,i}=\frac{1}{1+S_{b,k,i}}$$

TSSA output is achieved by applying gating and soft allocation to multi-head features:

(24) $$w_{b,k,l,i+1}=-\Pi_{b,k,l}G_{b,k,i}w_{b,k,l,i}$$

Finally, the outputs from all heads are concatenated and mapped back to the original dimension via a linear layer as the sub-layer’s output. By performing a low-rank projection on the sequence, it focuses on high-information features while ignoring low-information components, thereby reducing interference from unimportant information and enhancing the model’s performance.

### 2.4. LTiT: LSTM-TSSA-iTransformer

To more effectively capture global dependencies within subway section passenger flow sequences, we introduce the iTransformer encoder architecture. It treats independent time series as individual tokens, reverses the roles of the attention module and feedforward network (FFN), and leverages self-attention mechanisms to capture multivariate correlations.

iTransformer adopts an encoder-only architecture. $Z^{\left(0\right)}\leftarrow \mathrm{TSSA}(Z^{\left(0\right)})$ input encoder obtained after adaptive token adjustment via the TSSA, E layers are stacked, with the j th layer input being $Z^{\left(j-1\right)}\in {\mathbb{R}}^{B\times N\times d}$. The process is as follows:

Firstly, perform a linear projection on each head to obtain:

(25) $$Q=Z^{\left(j-1\right)}W_{Q},\;K=Z^{\left(j-1\right)}W_{K},\;V=Z^{\left(j-1\right)}W_{V}$$

Traditional self-attention mechanisms compute attention weights by calculating scaled dot products:

(26) $$\mathrm{Attn}(Q,K,V)=\mathrm{softmax}\left(\frac{QK^{⊤}}{\sqrt{d_{n}}}\right)V$$

In this model, the TSSA module replaces the traditional self-attention weight calculation (see Section 2.3 for the specific computation process). By computing the second-order moment statistics of tokens, it generates a weighted representation for each token. The attention weights for each head are then adjusted through gating and softmax mechanisms.

Each token undergoes a nonlinear transformation through a shared two-layer feedforward network:

(27) $$\mathrm{FFN}(o)=W_{2}\phi (W_{1}o+b_{1})+b_{2}$$

Combined with residual connections and LayerNorm to stabilize training:

(28) $${\tilde{Z}}^{\left(l\right)}=\mathrm{LayerNorm}\left(Z^{\left(l-1\right)}+MHA(Z^{\left(l-1\right)})\right)$$

(29) $$Z^{\left(l\right)}=\mathrm{LayerNorm}\left({\tilde{Z}}^{\left(l\right)}+\mathrm{FFN}({\tilde{Z}}^{\left(l\right)})\right)$$

Ultimately obtain the encoder output $Z^{\left(E\right)}\in {\mathbb{R}}^{B\times N\times d}$.

## 3. Experiment

### 3.1. Data Preparing

The data was sourced from Changsha Metro’s train operation schedules and entry/exit records captured by the city’s AFC system (including single-trip tickets, physical cards, and online tickets). Time span is from 15 October 2023 to 23 December 2023, including 6 lines and 135 stations, covering 18 transfer stations. The data includes ‘Entry Station’, ‘Entry Time’, ‘Card Number’, ‘Exit Station’, and ‘Exit Time’. To ensure data quality and the reliability of inferences, we preprocess the raw AFC records by removing entries with the same entry and exit stations, as well as outliers with travel times shorter than 5 min or longer than 180 min. This avoids the interference caused by incorrect card usage, data noise and atypical travel behaviors on the construction of the section passenger flow.

Based on the above data, we establish a section passenger flow inference model under normal operating conditions. Make the following fundamental assumptions:

1. Subway platforms have sufficient capacity to accommodate passengers entering, waiting and exiting, and there is no queue overflow caused by congestion.
2. The stop times of the subway are determined by the subway schedule and are not affected by the number of passengers boarding and alighting.
3. Passengers on the same subway are distributed evenly throughout the carriages.
4. When passengers get on, wait and get off subway, there will be no additional delays caused by conflicts and evasive maneuvers.
5. Passengers will not engage in unnecessary travel by continuing their ride because of missing their destination stop.

Passengers who use single-trip tickets, physical cards, or online methods to enter the subway station, often have to walk a certain distance to reach/leave the platform. The walking time for this distance is different. We assume that the time passengers walk from the turnstiles to the subway platform follows a normal distribution, with a mean time of 2 min and a range of [1 min, 3 min].

When a passenger’s origin and destination stations are not on the same line, a transfer is required to complete the journey. Given that multiple feasible transfer options may exist for the same origin-destination pair, passengers may diverge in their choices due to incomplete information or minor differences in travel times. We establish the following transfer path selection rules under network topology and timetable constraints:

If the estimated total travel time difference between two alternative transfer options does not exceed 5 min (roughly corresponding to the time required to pass through one station including stops), passengers will choose the shortest estimated route 80% of the time and the alternative route 20% of the time. If the time difference between two options exceeds 5 min, the option with the shortest estimated travel time is selected by default. Furthermore, to enhance consistency between inferred routes and actual travel, the total time calculated from timetables is compared against the actual total time recorded by the AFC. When the discrepancy exceeds 10 min (equivalent to waiting for an additional bus), the current route match is deemed unreasonable. The system then selects the next shortest route and recalculates until the time difference meets the threshold constraint. Walking time within stations during transfers also exhibits randomness. This study assumes transfer walking time follows a normal distribution with mean x min and range $\left[\left(x-1\right)\;\min,\;\left(x+1\right)\;\min\right]$. Here, x is derived from the corresponding transfer passenger walking time provided by Amap (Gaode map, v15.07.1.2037). Based on the above assumptions and route selection rules, the overall passenger trajectory inference procedure is summarized in Algorithm 1.


**Algorithm 1.** The Framework of Passenger Trajectory Inference
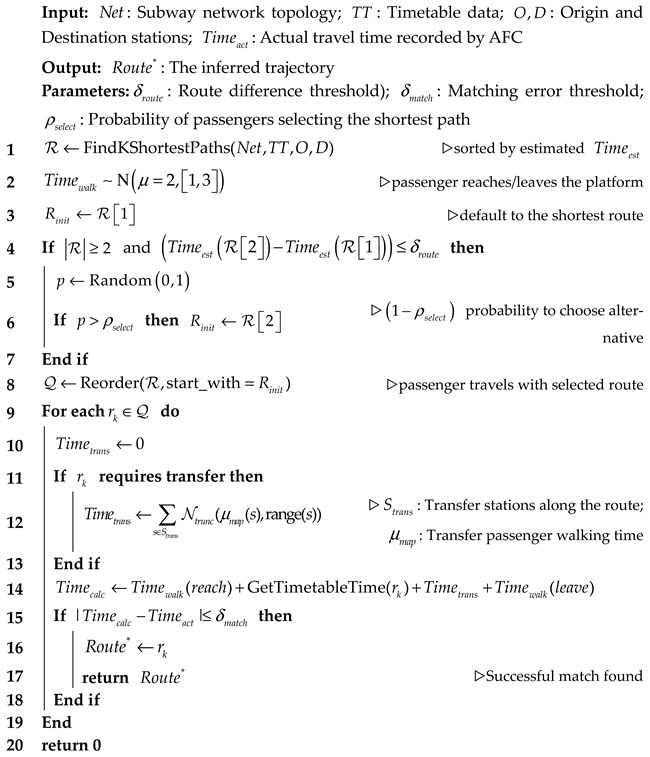
 


The external variables of temperature and precipitation data are sourced from the Environmental Meteorological Data Service Platform, eia-data.com (accessed on 2 January 2025).

Based on the above logic, we derive the subway section passenger flow. This study uses specific sections of Changsha Metro Line 1 as a case study for predictive analysis. The time step used for data acquisition corresponds to the 5-min interval.

### 3.2. Baseline Models and Experimental Setup

This section describes the experimental details and parameter settings of the model. All deep learning experiments were implemented in Python 3.12.12 using PyTorch 2.8.0 with CUDA 12.6 on the Kaggle platform, and were executed on a Tesla P100-PCIE-16GB GPU. The proposed model is compared with several fundamental traffic flow prediction models.

SARIMA [7]. The SARIMA model is formed by incorporating an additional seasonal term into the ARIMA model. The seasonal component of the model consists of terms similar to those in the non-seasonal part of the model, but with a shift in the seasonal cycle. This study employs the SARIMA (p, d, q) model, utilizing the Akaike Information Criterion (AIC) to determine the appropriate SARIMA order. All parameter estimates are obtained via maximum likelihood estimation, with only subway section passenger flow data input for forecasting when applying this model.

BP Neural Network [54]. The BP neural network is a multi-layer perceptron (MLP) model based on the gradient descent optimization algorithm. The model uses the backpropagation algorithm to adjust the weights within the network and effectively learn the nonlinear mapping relationship between inputs and outputs. It has good performance in dealing with complex time series data. The network structure consists of an input layer, a hidden layer, and an output layer. Hidden layer uses ReLU activation function, learning rate of Adam optimizer is set to 0.001, batch size is set to 64, and dropout rate is set to 0.2. The loss function for optimization is the mean squared error (MSE).

LightGBM [55]. LightGBM is an efficient implementation version of Gradient Boosting Decision Tree (GBDT), which relies on a histogram-based decision tree algorithm to quickly train on large-scale datasets. In this study, hyperparameters in LightGBM model mainly include maximum tree depth, learning rate, and number of leaf nodes. We use grid search and cross validation methods to select best hyperparameter configuration. The loss function for optimization is MSE.

LSTM [56]. LSTM is an improved variant of RNN, specifically designed to address the vanishing and exploding gradient issues. The model introduces gating mechanisms—including input gates, forget gates, and output gates—along with cell states. It makes LSTM selectively retain or forget information, and effectively capture long-term dependencies. In this study, the LSTM model is configured with two LSTM layers, each containing 128 hidden units. Dropout rate is set to 0.2 to prevent overfitting. The sigmoid activation function uses sigmoid in the gating mechanism and tanh in the candidate value calculation. The batch size is set to 64, utilizing the Adam optimizer with an initial learning rate of 0.001 and the ReduceLROnPlateau learning rate scheduling strategy.

EMAformer [57]: EMAformer is a deep learning model specifically designed for time-series forecasting tasks, incorporating temporal and cyclical information in its architecture. The model utilizes the attention mechanism combined with a special embedding strategy to capture long-term dependencies and seasonal patterns. EMAformer is configured with a two-layer encoder structure, where each layer has 64 hidden units. The model uses the GELU activation function and incorporates dropout layers with a 0.2 rate to prevent overfitting. The batch size is set to 64, the optimizer used is Adam with an initial learning rate of 0.001, and a ReduceLROnPlateau learning rate scheduler is employed for dynamic adjustment.

About the LTiT model, the LSTM layer consists of two layers with 128 hidden units, utilizing the GELU activation function and a dropout rate of 0.2 to prevent overfitting. The TSSA module uses 8 attention heads and a dropout rate of 0.2, with a learnable temperature parameter to adjust the attention weights. The iTransformer encoder contains 6 layers, each with 64 hidden units and a feedforward network size of 256, using GELU activation and a dropout rate of 0.1. The model is trained with a batch size of 64, using the Adam optimizer with an initial learning rate of 0.001, and a ReduceLROnPlateau learning rate scheduler for dynamic adjustment. External variables such as temperature and precipitation are embedded using the same dimensionality as the LSTM output to ensure consistency across the model.

### 3.3. Prediction

To compare the performance of different prediction models, the coefficient of determination (R^2^), mean absolute error (MAE), mean squared error (MSE), root mean squared error (RMSE) and mean absolute percentage error (MAPE) were employed to measure the deviation between predicted and actual subway section passenger flows.

(30) $$R^{2}=1-\sum_{t=1}^{n}{\left(y_{t}-{\hat{y}}_{t}\right)}^{2}/\sum_{t=1}^{n}{\left(y_{t}-\bar{y}\right)}^{2}$$

(31) $$MAE=\frac{1}{n}\sum_{t=1}^{n}\left|y_{t}-{\hat{y}}_{t}\right|$$

(32) $$MSE=\frac{1}{n}\sum_{t=1}^{n}{\left(y_{t}-{\hat{y}}_{t}\right)}^{2}$$

(33) $$RMSE=\sqrt{\frac{1}{n}\sum_{i=1}^{n}{\left(y_{i}-{\hat{y}}_{i}\right)}^{2}}]$$

(34) $$MAPE=\frac{1}{n}\sum_{t=1}^{n}\left|\frac{y_{t}-{\hat{y}}_{t}}{y_{t}}\right|\times 100\%$$

where $y_{t}$ represents the actual value at time t, ${\hat{y}}_{t}$ represents the predicted value at time t, and $\bar{y}$ is the average of the actual values. We use a 7:2:1 split for the dataset, where 70% of the data was allocated to training, 20% to validation, and 10% to testing. After implementing the training and testing process using the dataset, we present a comparative analysis of model errors between baseline models (SARIMA, BP neural network, LightGBM, LSTM, iTransformer, EMAformer) and ablation experiments (LSTM-iTransformer, TSSA-iTransformer), compared to the model developed in this study (LTiT).

Except for the SARIMA, all other models employ a one-step-ahead forecasting approach using the past 30 time points. Specifically, they predict the subway section passenger flow at the next 5-min time point based on the subway section passenger flow data from the preceding 150 min. The comparison results are shown in Table 1 and Table 2 for the high flow and low flow sections.

It can be observed that the LTiT model proposed in this paper achieves significant improvements over other baseline models in terms of evaluation metrics such as R^2^, MAE, RMSE, MSE, MAPE. The increase in R^2^ indicates that the model can more accurately fit the subway section passenger flow. Lower RMSE and MSE values indicate that the model exhibits smaller overall errors on subway section passenger flow prediction. The decrease in MAE means that model can still maintain low errors and effectively predict peak passenger flow with high passenger flow. The decrease in MAPE indicates that the model has smaller prediction errors and performs well during low passenger flow periods.

We can observe that, for the low flow section, the model’s prediction accuracy decreases compared to the high flow section. This decrease in accuracy can be attributed to the inherent unpredictability and high variability in passenger flow during low traffic periods. Passenger behavior in low flow conditions is more susceptible to noise and less influenced by predictable patterns such as daily commuting routines. Additionally, lower passenger volumes make it harder for models to capture meaningful patterns, leading to increased errors. However, despite this decrease in accuracy, the LTiT model still demonstrates the best performance across all models, achieving the highest R^2^, the lowest RMSE, MSE, MAE, and MAPE values. The EMAformer model exhibits high prediction accuracy in high-flow sections, but its accuracy significantly decreases in low-low sections. This may be due to the EMAformer model’s strong ability to predict time series with clear periodic patterns, while in low-traffic sections, the model’s performance is more affected by data noise, leading to a decline in accuracy. In contrast, the LTiT model is less susceptible to noise, and its performance remains relatively stable in low-flow sections. This stability is directly attributed to the TSSA module, which effectively suppresses the influence of random fluctuations by utilizing second-order moments, maintaining high prediction accuracy even in noisy, low-flow sections.

We conducted ablation experiments to validate the effectiveness of two modules within the model. The results showed that removing either the LSTM or TSSA module led to a decline. This indicates that the LSTM module effectively extracts temporal features from sequences, and that the TSSA module can also prevent the model from learning during low-information periods and capture high-information periods, thereby significantly improving model performance. For the low flow section, removing the TSSA module causes a sharp deterioration in the MAPE. It confirms that without TSSA’s adaptive second-order statistical weighting, the temporal features extracted by LSTM are easily contaminated by random noise.

We performed experiments that removed temperature, precipitation, and all external variables, and observed a notable drop in prediction accuracy in all cases. This further supports the importance of incorporating external variables in the model. Once external variables are removed, it will lead to a decrease in accuracy of model’s estimation. This result indicates that weather factors significantly influence subway section passenger flow prediction. When dealing with complex time series data, as these external variables can effectively capture the seasonal and environmental factors influencing passenger flow. These external variables provide additional contextual information, enabling the model to better adapt to real-world changes and improving predictive accuracy.

**Figure 4:**
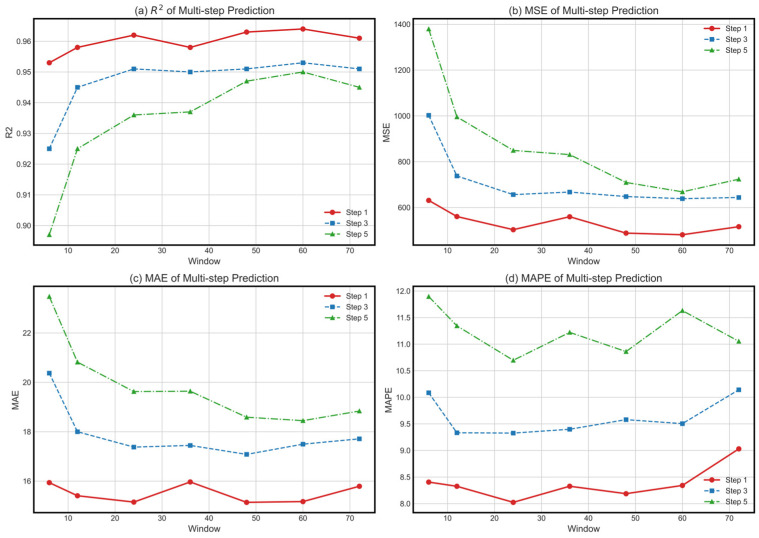
(**a**–**d**) display the variations of R^2^, MSE, MAE, and MAPE for the LTiT model of different prediction steps (1, 3, 5) as the input window length changes.

We further investigate the model’s performance under different input windows and prediction steps. Input windows were set to the past 6, 12, 24, 36, 48, 60, and 72 time points. Prediction steps were set to 1, 3, and 5 time points for comparative experiments. That is to say, we used historical data from the past 30, 60, 120, 180, 240, 300, and 360 min to forecast future subway section passenger flows at 1, 3, and 5 prediction steps. The comparison results are shown in Table 3 and Figure 4.

In single-step prediction tasks, appropriately lengthening the input windows can improve model performance. This shows that longer historical windows provide more information for the model, helping it better capture short-term trends and periodic characteristics. Longer input window length enables the model to cover more traffic flow patterns over extended periods (such as peak hours and commuting times) and provides sufficient information to predict future subway section passenger flow. If input window length is too long, it will introduce too much irrelevant information, leading to a decline in model accuracy.

In multi-step prediction tasks, errors gradually propagate. The selection of window length increasingly impacts model accuracy. Evaluation metrics show that if step size is increased, trend of reduction will become more pronounced. If input window length is too long, it will dilute the key features learned by the model. It will result in a low evaluation rate and difficulty in capturing most significant information for future predictions. When the input window length is set to 60, the model performs best. This is achieved by using subway section passenger flow data from the past 300 min to predict the flow at the next time point.

To simulate the uncertainty of real-world data, we first train the model on a noise-free training set, then inject noise into the test set. This helps us evaluate the model’s performance when facing different types of noise. We selected three common types of noise: Gaussian noise, impulse noise, and missing data. The purpose of adding Gaussian noise to the data is to simulate random and uniformly distributed errors. Impulse noise typically manifests as sudden, large values that appear randomly. This type of noise often simulates outliers or extreme erroneous inputs in the data, which may occur due to sensor errors or data input failures. Missing data is another common issue, especially in real-world applications where data can be missing for various reasons. Therefore, introducing missing values during testing allows us to assess the model’s robustness in handling incomplete data.

**Figure 5:**
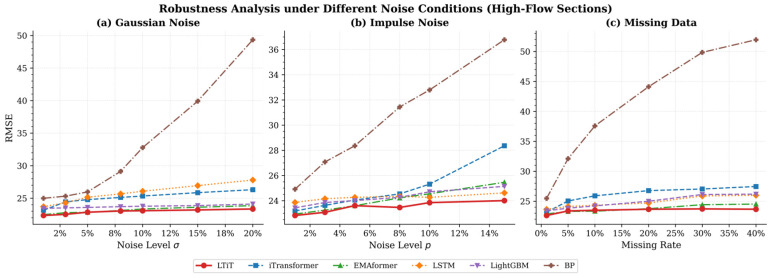
Robustness Analysis under Different Noise Conditions (High-Flow Sections).

**Figure 6:**
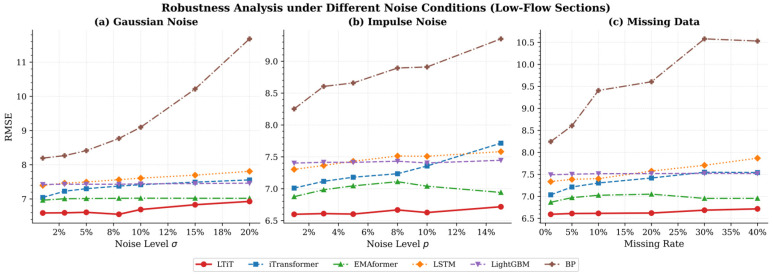
Robustness Analysis under Different Noise Conditions (Low-Flow Sections).

Figure 5 and Figure 6 show the robustness of different models under Gaussian noise, impulse noise, and missing data noise. The models contains LTiT, iTransformer, EMAformer, LSTM, LightGBM, BP. The evaluation metric used is RMSE. A lower RMSE value, and a flatter curve as the noise level increases, indicates stronger interference resistance and robustness of the model. In all test scenarios and under both high-flow sections and low-flow sections, the LTiT model consistently demonstrated the best performance. The BP neural network was extremely sensitive, and its RMSE deteriorating exponentially as the noise level increased. Models such as iTransformer and LSTM showed some resistance, but the error still exhibited a gradual upward trend. LTiT, EMAformer, and LightGBM exhibited good stability, with LTiT remaining consistently at the optimal level. When facing impulse noise, iTransformer shows a noticeable increase in error. The traditional attention mechanism’s softmax function is susceptible to impulse noise, causing the attention weights to be disproportionately amplified by sudden noise, which in turn affects the prediction results. By introducing the TSSA attention mechanism, the impact of such sudden noise can be effectively reduced, thereby enhancing the model’s robustness. The relative rankings and trends of all models under high/low flow conditions remained highly consistent, further confirming the universality of the conclusions. Overall, the experimental data clearly demonstrate that the LTiT model possesses the best generalization and interference resistance capabilities. The traditional BP model struggles to handle imperfections in real-world data, while iTransformer and LightGBM are also vulnerable under certain types of noise, such as impulse noise.

## 4. Discussion

After defining the subway section passenger flow prediction problem and establishing the overall prediction framework, we present the implementation details of LTiT. We then conducted comparative experiments between LTiT and other baseline models. Ablation studies and sensitivity analyses examining different input window sizes and prediction lengths were also conducted. Based on comprehensive experimental results, following conclusion can be drawn:

- Comparison with Baseline Models: Compared to baseline methods such as SARIMA, BP neural networks, LightGBM, LSTM, and iTransformer, LTiT achieved best performance in metrics including R^2^, RMSE, MSE, MAE, and MAPE. This shows that the model can more accurately capture the true changes of subway section passenger flow, while reflecting lower errors and higher stability.
- Experimental Validation of Module Effectiveness: We compared LSTM-iTransformer and TSSA-iTransformer with the full model LTiT. Results show that if we incorporate LSTM to capture the temporal characteristics of subway section passenger flow and introduce TSSA for adaptive weighting the model will perform better. The combination of these two parts further improves prediction accuracy, showing that different modules complement each other in capturing temporal characteristics and modeling global relationships.
- Different Input Windows and Prediction Steps: In sensitivity experiments, we select past time points at 6, 12, 24, 36, 48, 60, and 72 as input windows to predict future time points at 1, 3, and 5 as prediction steps. The results show that as the input window lengthens, the model demonstrates a greater ability to capture temporal features. However, excessively long input windows may introduce increased noise and redundant information. As the prediction step size extends, the model experiences cumulative error, resulting in a decrease in model accuracy. The model still performs well in accomplishing the prediction tasks.
- Robustness Analysis under Different Noise Conditions: We perform robustness analysis with six models under Gaussian noise, impulse noise, and missing data. Results show that LTiT outperforms other models in both high-flow and low-flow sections, exhibiting strong robustness with a stable RMSE across noise levels. The BP network is highly sensitive to noise, with its RMSE worsening exponentially. iTransformer and LSTM showed some resistance, but errors still increased gradually. The introduction of the TSSA attention mechanism in iTransformer significantly improved its robustness, particularly against impulse noise. LTiT demonstrated the best generalization and interference resistance capabilities.

Subway section passenger flow predictions are influenced by multiple factors. Subway section passenger flow exhibits strong nonlinearity and temporal dependence. During weekdays, it shows significant peaks during morning and evening commuting hours. On weekends and holidays, overall passenger flow increases, while fluctuations become more continuous and dispersed. Relying on linear models (such as SARIMA) or shallow machine learning models often proves challenging for capturing such non-stationary and nonlinear dynamic changes. This is also why SARIMA performs worst. The LSTM module can use gated memory mechanism to capture temporal characteristics of subway section passenger flow within a limited time window. This provides the iTransformer with smoother, higher-order feature representations, which can help the model to learn periodic characteristics and cross-temporal relationships over longer timescales.

Not all time points in the subway section passenger flow sequence contribute equally to the prediction. Peak periods and their surrounding times often provide more information, while low-traffic periods are more easily affected by noise. Following embedding, we introduce a TSSA-inspired variant attention module. This applies adaptive weighting to token representations at each time step, enhancing high-information segments and suppressing noise. This improves the learning efficiency of iTransformer. Combining both approaches can further enhance the model’s accuracy.

Our ablation experiments and robustness analysis confirm that simply combining LSTM and iTransformer can help extract time features, but this is still not enough for the noisy environment of section passenger flow. The key value of the LTiT model lies in the targeted introduction of the TSSA module. TSSA module plays an important role in the whole model. As demonstrated in our ablation experiments, traditional attention methods do not work well under high-noise subway sections, whereas the TSSA’s second-order statistical weighting successfully filters this noise. This proves that our specific architectural enhancement directly resolves the inherent limitations of standard LSTM-iTransformer hybrids in this task.

## 5. Conclusions

Current passenger flow prediction research primarily focuses on short-term inbound/outbound station traffic prediction or short-term subway OD demand prediction. Subway section passenger flow prediction can improve existing prediction systems, assisting subway operators in monitoring real-time passenger load conditions in carriages.

We divided the subway section passenger flow prediction task into two parts: data preprocess and prediction model. During the data preprocessing part, we combined AFC data with subway train schedules to derive subway section passenger flows. During the prediction modeling part, we designed an LTiT model to effectively capture the temporal characteristics and global dependencies within passenger flow data, thereby enhancing prediction accuracy.

To validate the effectiveness of the proposed model, we compared it with baselines. Experimental results show that the LTiT model achieves better performance on various evaluation metrics, including R^2^, RMSE, MSE, MAE, and MAPE. It outperforms traditional SARIMA, BP neural networks, and other machine learning models, particularly in capturing complex temporal features and nonlinear relationships. In different input windows and prediction steps, the model shows excellent predictive capabilities.

It should be noted that the estimated subway section passenger flow is derived from AFC data and subway schedules. Our passenger flow inference model relies on a simplified assumption for route selection, assigning a static 80/20 probability split between the shortest and alternative routes. We acknowledge that real-world passenger route-choice behavior is far more complex and heavily influenced by dynamic factors such as station crowding, transfer conditions, and seat availability. Due to current data limitations, these human behavioral factors were not fully integrated into the model. Furthermore, It is difficult to obtain actual passenger flow data for comparison. This will require subway operators to further validate its accuracy through methods such as subway load weight measurements or video monitors. Future research could expand the model’s applicability by incorporating more external variables—such as holidays and special events—to account for their impact on traffic flow.

## Figures and Tables

**Table 1:** Comparison of Prediction Results Between LTiT Model and Baseline Model, and Comparison of Prediction Results from Ablation Experiments (high flow section).

Model	R^2^	MSE	RMSE	MAE	MAPE
SARIMA	0.893	1247.86	35.325	19.256	14.253
BP Neural Network	0.932	620.830	24.916	18.084	11.138
LightGBM	0.952	535.700	23.145	15.875	8.532
LSTM	0.948	557.990	23.622	16.835	11.436
iTransformer	0.955	532.847	23.084	15.626	8.335
EMAformer	0.962	507.416	22.526	15.025	7.968 *
LSTM-iTransformer	0.959	527.261	22.963	15.264	8.246
TSSA-iTransformer	0.960	526.343	22.943	15.425	8.433
LTiT	0.964 *	498.002 *	22.316 *	14.957 *	8.028
LTiT (without temperature)	0.947	557.564	23.613	17.796	9.350
LTiT (without precipitation)	0.950	546.068	23.368	17.266	9.236
LTiT (without external variables)	0.935	601.048	24.516	18.603	9.789

* The best results are highlighted in bold and marked with ‘*’. Units for Evaluation Metrics: MSE: Passengers^2^; RMSE: Passengers; MAE: Passengers; MAPE: Percentage (%).

**Table 2:** Comparison of Prediction Results Between LTiT Model and Baseline Model, and Comparison of Prediction Results from Ablation Experiments (low flow section).

Model	R^2^	MSE	RMSE	MAE	MAPE
SARIMA	0.621	149.705	12.235	8.231	41.254
BP Neural Network	0.812	63.125	7.945	4.937	27.767
LightGBM	0.844	55.534	7.452	4.770	23.408
LSTM	0.854	53.599	7.321	4.744	25.739
iTransformer	0.895	48.109	6.936	4.433	17.923
EMAformer	0.896	47.848	6.917	4.083	17.595
LSTM-iTransformer	0.912	43.624	6.567	4.189	17.783
TSSA-iTransformer	0.904	43.703	6.611	4.213	16.556
LTiT	0.918 *	43.474 *	6.594 *	4.027 *	15.803 *
LTiT (without temperature)	0.894	48.387	6.956	4.468	18.459
LTiT (without precipitation)	0.889	49.661	7.047	4.547	19.805
LTiT (without external variables)	0.877	52.407	7.239	4.677	20.561

* The best results are highlighted in bold and marked with ‘*’. Units for Evaluation Metrics: MSE: Passengers^2^; RMSE: Passengers; MAE: Passengers; MAPE: Percentage (%).

**Table 3:** Performance of the LTiT Model Across Different Prediction Step Sizes and Input Window Lengths (high-flow section as an example).

Step	Window	R^2^	RMSE	MSE	MAE	MAPE
1	6	0.953	25.121	631.079	15.937	8.405
	12	0.958	23.677	560.593	15.406	8.326
	24	0.962	22.433	503.222	15.150	8.024 *
	36	0.958	23.659	559.736	15.965	8.327
	48	0.963	22.094	488.151	15.139 *	8.187
	60	0.964 *	21.933 *	481.072 *	15.170	8.342
	72	0.961	22.720	516.221	15.790	9.030
3	6	0.925	31.662	1002.464	20.374	10.086
	12	0.945	27.160	737.686	17.999	9.333
	24	0.951	25.617	656.250	17.377	9.327 *
	36	0.950	25.834	667.405	17.444	9.399
	48	0.951	25.452	647.813	17.082 *	9.580
	60	0.953 *	25.269 *	638.542 *	17.495	9.505
	72	0.951	25.374	643.822	17.711	10.142
5	6	0.897	37.140	1379.377	23.474	11.896
	12	0.925	31.552	995.555	20.819	11.345
	24	0.936	29.140	849.132	19.626	10.697 *
	36	0.937	28.826	830.959	19.641	11.222
	48	0.947	26.633	709.333	18.587	10.861
	60	0.950 *	25.852 *	668.319 *	18.448 *	11.632
	72	0.945	26.899	723.529	18.837	11.054

* The best results are highlighted in bold and marked with ‘*’. Units for Evaluation Metrics: MSE: Passengers^2^; RMSE: Passengers; MAE: Passengers; MAPE: Percentage (%).

## Data Availability

The data are available from the corresponding author on reasonable request.
